# Docosahexaenoic Acid Suppresses Oxidative Stress-Induced Autophagy and Cell Death via the AMPK-Dependent Signaling Pathway in Immortalized Fischer Rat Schwann Cells 1

**DOI:** 10.3390/ijms23084405

**Published:** 2022-04-15

**Authors:** Yasuaki Tatsumi, Ayako Kato, Naoko Niimi, Hideji Yako, Tatsuhito Himeno, Masaki Kondo, Shin Tsunekawa, Yoshiro Kato, Hideki Kamiya, Jiro Nakamura, Koji Higai, Kazunori Sango, Koichi Kato

**Affiliations:** 1 Laboratory of Medicine, Aichi Gakuin University School of Pharmacy, 1-100 Kusumoto-cho, Chikusa-ku, Nagoya 464-8650, Japan; ytatsumi@dpc.agu.ac.jp (Y.T.); k-ayako@dpc.agu.ac.jp (A.K.); 2 Department of Medical Biochemistry, Faculty of Pharmaceutical Sciences, Toho University, Miyama 2-2-1, Funabashi 274-8510, Japan; koji@phar.toho-u.ac.jp; 3 Diabetic Neuropathy Project, Department of Diseases and Infection, Tokyo Metropolitan Institute of Medical Science, 2-1-6 Kamikitazawa, Setagaya, Tokyo 156-8506, Japan; niimi-nk@igakuken.or.jp (N.N.); yako-hd@igakuken.or.jp (H.Y.); sango-kz@igakuken.or.jp (K.S.); 4 Division of Diabetes, Department of Internal Medicine, Aichi Medical University School of Medicine, 1-1 Yazakokarimate, Nagakute 480-1195, Japan; thimeno@aichi-med-u.ac.jp (T.H.); kondou.masaki.330@mail.aichi-med-u.ac.jp (M.K.); tsune87@aichi-med-u.ac.jp (S.T.); ykato4@aichi-med-u.ac.jp (Y.K.); hkamiya@aichi-med-u.ac.jp (H.K.); jiro@aichi-med-u.ac.jp (J.N.)

**Keywords:** DHA, oxidative stress, autophagy, AMPK, Schwann cells

## Abstract

Autophagy is the process by which intracellular components are degraded by lysosomes. It is also activated by oxidative stress; hence, autophagy is thought to be closely related to oxidative stress, one of the major causes of diabetic neuropathy. We previously reported that docosahexaenoic acid (DHA) and eicosapentaenoic acid (EPA) induced antioxidant enzymes and protected Schwann cells from oxidative stress. However, the relationship between autophagy and oxidative stress-induced cell death in diabetic neuropathy has not been elucidated. Treatment with tert-butyl hydroperoxide (tBHP) decreased the cell survival rate, as measured by an MTT assay in immortalized Fischer rat Schwann cells 1 (IFRS1). A DHA pretreatment significantly prevented tBHP-induced cytotoxicity. tBHP increased autophagy, which was revealed by the ratio of the initiation markers, AMP-activated protein kinase, and UNC51-like kinase phosphorylation. Conversely, the DHA pretreatment suppressed excessive tBHP-induced autophagy signaling. Autophagosomes induced by tBHP in IFRS1 cells were decreased to control levels by the DHA pretreatment whereas autolysosomes were only partially decreased. These results suggest that DHA attenuated excessive autophagy induced by oxidative stress in Schwann cells and may be useful to prevent or reduce cell death in vitro. However, its potentiality to treat diabetic neuropathy must be validated in in vivo studies.

## 1. Introduction

In diabetic patients, long-term hyperglycemia causes severe diabetic complications, such as neuropathy, nephropathy, and retinopathy [1]. In particular, diabetic peripheral neuropathy is associated with pain and numbness, impairing life quality [2]. Diabetic neuropathy causes severe damage to the peripheral nerve function through various mechanisms, such as the polyol pathway, advanced glycation end-product production, the activation of protein kinase C, and oxidative stress [3]. All these pathways cause mitochondrial dysfunction and promote reactive oxygen species (ROS) accumulation, which further results in neural cell damage and contributes to the development and progression of diabetic neuropathy [4]. We have shown that apoptosis and ROS-mediated endoplasmic reticulum (ER) stress responses were induced by recurrent short-term hyperglycemia in immortalized adult mouse Schwann (IMS32) cells [5]. We focused on oxidative stress as one of the important factors underlying diabetic neuropathy pathogenesis. tert-butyl hydroperoxide (tBHP) is widely used to induce oxidative stress in in vitro studies on diabetes and its complications [6,7,8]. tBHP induced intracellular ROS and cell death in IMS32 cells in our previous study [9]. Therefore, we used tBHP in the present study. The immortalized Fisher rat Schwann cells 1 (IFRS1) line was established by Sango et al. [10]. Unlike IMS32 cells, IFRS1 cells exhibit a fundamental ability to myelinate axons in a co-culture with dorsal root ganglion neurons [10]. Moreover, IFRS1 cells have been used as model cells for impaired autophagy associated with neuropathy induced by amiodarone [11]. Therefore, IFRS1 cells are a useful tool for analyzing autophagy induced by oxidative stress.

The physiological effects of n-3 polyunsaturated fatty acids (n-3 PUFAs), such as docosahexaenoic acid (DHA), are known to have antioxidant [12], anti-inflammatory [13], cardiovascular-protective [14], and neuroprotective effects [15]. We recently reported that DHA and eicosapentaenoic acid (EPA) induced antioxidant enzymes—including heme oxygenase-1 (Ho-1), NAD(P)H:quinone oxidoreductase-1 (Nqo1), and catalase—through a nuclear factor erythroid 2-related factor 2 (Nrf2) transcript and protected IMS32 cells from oxidative stress [9]. This result suggests that DHA and EPA could be used as treatments for diabetic neuropathy based on their pathogenic mechanisms.

A pretreatment with DHA caused a transient increase in cellular ROS levels that activated Nrf2 and p62 and induced the selective autophagy of impaired proteins by inducing endogenous antioxidants in human retinal pigment epithelial cells [16]. These results suggest that DHA might regulate autophagy to reduce the risk of cell damage. On the other hand, DHA effectively reduced TNF-α-caused necroptosis and autophagy by attenuating ROS production, ceramide production, lysosomal impairment, and cathepsin L activation in the L929 murine fibrosarcoma cell line [17]. This result suggests that DHA reduces TNF-α-induced necroptosis and autophagy. Accordingly, DHA has also been shown to have dual effects on autophagy, depending on the cellular environment.

Autophagy is a catabolic process that removes damaged intracellular organelle components by lysosomal degradation [18]. Autophagy has been reported to be activated in response to cellular stress, such as ROS [19]. Hyperglycemia-induced autophagy is also mediated through ROS generation. Defective autophagy in pancreatic β-cells has been reported at the onset of type 2 diabetes [20], showing that autophagy plays an important role in maintaining the β-cell function [21,22,23]. Autophagy dysregulation is associated with obesity, insulin resistance, and impaired glucose tolerance [24]. Additionally, autophagy induced by high glucose has been reported to be mediated by ROS generation in podocytes [25,26,27,28]. The induction of autophagy has been reported by the exposure of serum from type 2 diabetic patients with neuropathy to human neuroblastoma SH-SY5Y cells [29], suggesting a possible autophagy progression in diabetic neuropathy.

However, little research has been undertaken on the relationship between autophagy and diabetic neuropathy. Furthermore, it remains unclear whether DHA protects Schwann cells via the autophagy pathway. Therefore, we evaluated the influence of oxidative stress on the cell survival rate and autophagy signal transduction and examined the effect of DHA on oxidative stress-induced autophagy in Schwann cells.

## 2. Results

### 2.1. DHA Protects tBHP-Induced Cytotoxicity in the IFRS1 Cell Line

**Figure 1:**
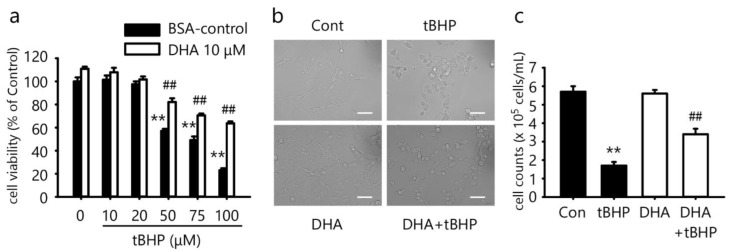
Docosahexaenoic acid (DHA) protects against tert-butyl hydroperoxide (tBHP)-induced cytotoxicity in immortalized Fischer rat Schwann cells 1 (IFRS1). (**a**) Treatment with tBHP for 3 h decreased cell survival rate measured by MTT assay in IFRS1 cells whereas pretreatment with 10 μM DHA for 12 h significantly protected against tBHP-induced cytotoxicity. Each value represents the mean ± S.E. of six experiments. **: *p* < 0.01 compared with the BSA-control; ^##^: *p* < 0.01 compared with each tBHP concentration. (**b**) Cell morphology examined by phase-contrast microscopy. Scale bar: 50 μM. (**c**) Cell counts examined by microscopy. Each value represents the mean ± S.E. of three experiments. **: *p* < 0.01 compared with the BSA-control; ^##^: *p* < 0.01 compared with each tBHP concentration.

We previously reported that DHA protected IMS32 cells from oxidative stress [9]. Therefore, in this study, we investigated whether DHA protected against oxidative stress-induced cytotoxicity in IFRS1 cells. To determine whether DHA could prevent cell death in IFRS1 cells treated with tBHP, an MTT assay was used as previously described [9]. A treatment with 10–100 μM tBHP for 3 h decreased the cell viability in a dose-dependent manner (Figure 1a). A pretreatment with 10 μM DHA for 12 h significantly protected the cells from tBHP-induced cytotoxicity (Figure 1a). The control cells and the cells pretreated with 10 μM DHA retained a spindle-shaped morphology. Most of the cells treated with 50 μM tBHP for 3 h shrank in size and were less abundant (Figure 1b). A pretreatment with 10 μM DHA for 12 h protected against cell morphology damage by tBHP (Figure 1b). We also counted the number of cells to measure cell death. A treatment with 50 µM tBHP significantly reduced the number of cells (Figure 1c). A pretreatment with 10 μM DHA increased the number of cells, which was decreased by tBHP (Figure 1c). These results demonstrated that tBHP induced cell death and a pretreatment with DHA efficiently protected against oxidative stress-induced cytotoxicity in IFRS1 cells.

### 2.2. DHA Suppresses tBHP-Induced ROS Production in the IFRS1 Cell Line

**Figure 2:**
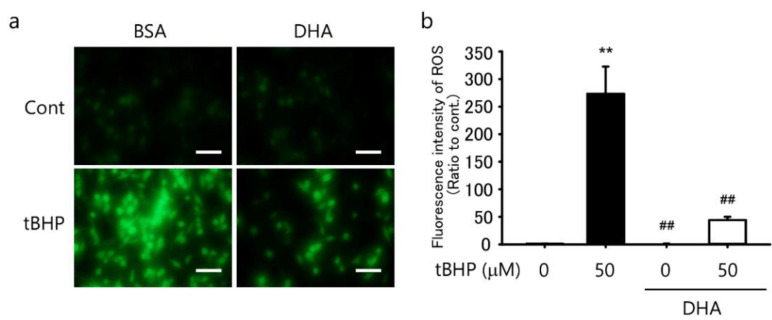
Docosahexaenoic acid (DHA) suppressed tert-butyl hydroperoxide (tBHP)-induced intracellular reactive oxygen species (ROS) in immortalized Fischer rat Schwann cells 1 (IFRS1). (**a**) Treatment with tBHP for 30 min induced ROS production in IFRS1 cells as monitored by fluorescence microscopy whereas pretreatment with 10 μM DHA for 12 h significantly suppressed tBHP-induced ROS production. Scale bar: 100 μM. (**b**) Quantification of fluorescent intensity of ROS in IFRS1 cells. Each value represents the mean ± S.E. of eight experiments. **: *p* < 0.01 compared with the control; ^##^: *p* < 0.01 compared with the 50 μM tBHP control.

We previously reported that DHA suppressed ROS production in IMS32 cells [9]. Therefore, in the present study, we investigated whether DHA also suppressed ROS production in IFRS1 cells. A treatment with 50 μM tBHP for 30 min induced intercellular ROS production (Figure 2a,b) whereas a pretreatment with 10 μM DHA for 12 h significantly suppressed ROS production (Figure 2a,b). These results demonstrated that a pretreatment with DHA efficiently suppressed tBHP-induced intracellular ROS production in IFRS1 cells.

### 2.3. tBHP Induces Autophagy in the IFRS1 Cell Line

**Figure 3:**
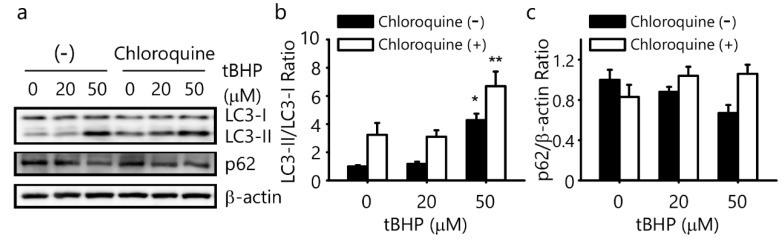
Tert-butyl hydroperoxide (tBHP) induces autophagy in immortalized Fischer rat Schwann cells 1 (IFRS1). (**a**–**c**) IFRS1 cells treated with different concentrations of tBHP in the presence or absence of 25 μM chloroquine for 3 h were analyzed by Western blotting. Each value represents the mean ± S.E. of three experiments. *: *p* < 0.05 and **: *p* < 0.01 compared with the control. LC3: microtubule-associated protein 1 light chain 3.

To evaluate the tBHP-induced signal transduction of autophagy in IFRS1 cells, Western blotting was used to quantify the microtubule-associated protein 1 light chain 3 (LC3)-II/LC3-I ratio and protein expression of p62. A 50 μM tBHP treatment significantly increased the LC3-II/LC3-I ratio whereas the expression of p62 protein decreased in response to a 50 μM tBHP treatment in IFRS1 cells (Figure 3a–c). To evaluate autophagic flux, the IFRS1 cells were treated with tBHP in the presence of chloroquine, which inhibits autophagosome degradation. The chloroquine treatment significantly increased the LC3-II/LC3-I ratio, which was increased by 50 μM tBHP, and increased the p62 protein expression, which was decreased by 50 μM tBHP in the IFRS1 cells (Figure 3a–c). These results indicated that oxidative stress induced by 50 μM tBHP may promote autophagy at the first step of autophagy initiation; i.e., autophagosome formation.

### 2.4. DHA Suppresses tBHP-Induced Autophagy in the IFRS1 Cell Line

**Figure 4:**
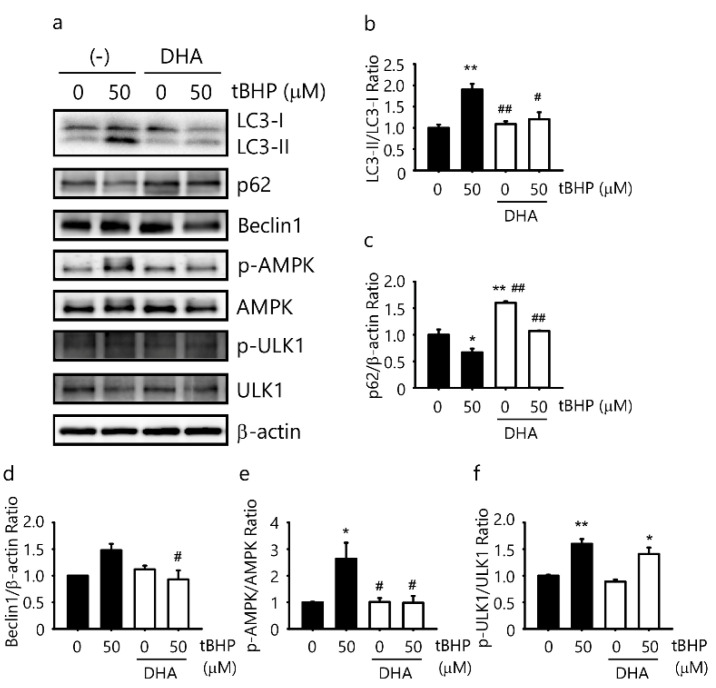
Docosahexaenoic acid (DHA) suppresses tert-butyl hydroperoxide (tBHP)-induced autophagy in immortalized Fischer rat Schwann cells 1 (IFRS1). (**a**–**f**) IFRS1 cells treated with 50 μM tBHP for 3 h in the presence or absence of pretreated 10 μM DHA were analyzed by Western blotting. Each value represents the mean ± S.E. of three experiments. *: *p* < 0.05 and **: *p* < 0.01 compared with the control; ^#^: *p* < 0.05 and ^##^: *p* < 0.01 compared with the 50 μM tBHP control. LC3: microtubule-associated protein 1 light chain 3; AMPK: AMP-activated protein kinase; ULK1: UNC51-like kinase.

To investigate whether DHA could attenuate tBHP-induced autophagy signal transduction in IFRS1 cells, Western blotting was used to quantify the LC3-II/LC3-I ratio and p62 protein expression. A 50 μM tBHP treatment increased the LC3-II/LC3-I ratio and decreased the protein expression of p62 (Figure 4a–c). To gain a further insight into autophagy signal transduction, we assessed the AMP-activated protein kinase/UNC51-like kinase (AMPK/ULK1) pathway and Beclin1. A 50 μM tBHP treatment increased AMPK phosphorylation at Thr172, ULK1 phosphorylation at Ser317, and protein expression of Beclin1 in the IFRS1 cells (Figure 4a,d–f). Additionally, the pathway for tBHP-induced autophagy was evaluated by a pretreatment with 10 μM DHA for 12 h. A pretreatment with 10 μM DHA for 12 h significantly decreased the LC3-II/LC3-I ratio, which was elevated by tBHP. A DHA pretreatment increased the p62 protein expression, which was decreased by tBHP, and decreased AMPK phosphorylation at Thr172 as well as the Beclin1 protein expression, which was increased by tBHP in the IFRS1 cells (Figure 4a–f). However, ULK1 phosphorylation at Ser317 tended to decrease following a DHA pretreatment (Figure 4a,f). These results indicate that a DHA pretreatment suppresses tBHP-induced autophagy via the AMPK signaling pathway (partly through ULK1) by blocking the initiation of the autophagic process.

### 2.5. AMPK Inhibitor Protects tBHP-Induced Cytotoxicity in the IFRS1 Cell Line

**Figure 5:**
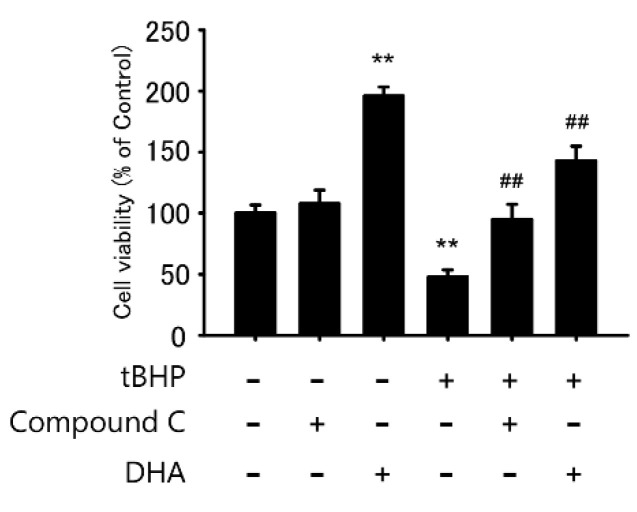
AMP-activated protein kinase (AMPK) inhibitor Compound C protects against tert-butyl hydroperoxide (tBHP)-induced cytotoxicity in immortalized Fischer rat Schwann cells 1 (IFRS1). Treatment with tBHP for 3 h decreased survival rate in IFRS1 cells as measured by MTT assay whereas pretreatment with 5 μM Compound C or 10 μM DHA for 12 h significantly protected against tBHP-induced cytotoxicity. Each value represents the mean ± S.E. of six experiments. **: *p* < 0.01 compared with tBHP(-); ^##^: *p* < 0.01 compared with tBHP(+).

To evaluate whether the AMPK pathway was involved in oxidative stress-induced cell death in IFRS1 cells, we examined if the administration of Compound C, an AMPK inhibitor, protected against oxidative stress-induced cytotoxicity in IFRS1 cells. A pretreatment with 5 μM Compound C for 12 h significantly protected the cells from tBHP-induced cytotoxicity (Figure 5). This result demonstrated that a pretreatment with Compound C efficiently protected against oxidative stress-induced cytotoxicity in the IFRS1 cells, as observed in response to a DHA treatment, indicating that the effect was mediated via the AMPK signaling pathway.

### 2.6. DHA Suppresses tBHP-Induced Autophagosomes in the IFRS1 Cell Line

**Figure 6:**
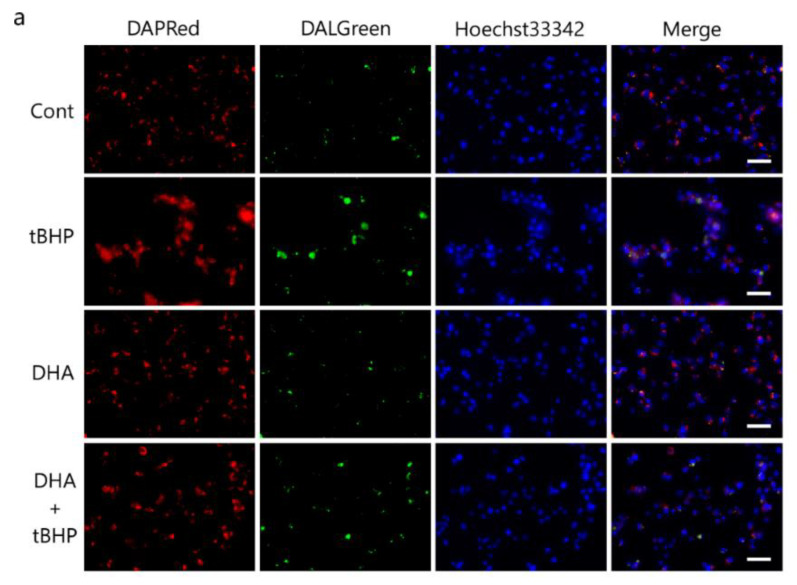

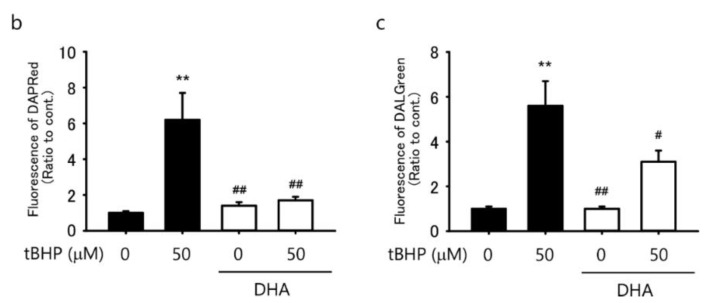
Docosahexaenoic acid (DHA) suppresses tert-butyl hydroperoxide (tBHP)-induced autophagosomes and autolysosomes in immortalized Fischer rat Schwann cells 1 (IFRS1). Autophagy levels induced by 50 μM tBHP in IFRS1 cells were revealed by DAPRed (autophagosome (**a**,**b**)) and DALGreen staining (autolysosome (**a**,**c**)). Each value represents the mean ± S.E. of eight experiments. **: *p* < 0.01 compared with the control; ^##^: *p* < 0.01 and ^#^: *p* < 0.05 compared with the 50 μM tBHP control. Scale bar: 50 μM.

We then investigated whether DHA reduced oxidative stress-induced autophagosomes and autolysosomes. DAPRed and DALGreen staining as well as fluorescent probes for monitoring the formation of autophagosomes and autolysosomes [30] showed that both autophagosomes (Figure 6a,b) and autolysosomes (Figure 6a,c) were induced by 50 μM tBHP in IFRS1 cells. The formation of tBHP-induced autophagosomes decreased to control levels by a DHA pretreatment whereas the levels of autolysosomes were only partially decreased (Figure 6a–c). These results suggest that a DHA pretreatment suppresses the tBHP-induced autophagy process shown by autophagosomes and autolysosomes.

## *3.* Discussion

This is the first study to report the potential of DHA in suppressing oxidative stress-induced autophagy and protecting Schwann cells from death induced by oxidative stress. In the IFRS1 cell line, tBHP caused autophagy and a DHA pretreatment suppressed tBHP-induced autophagy. In addition, autophagosomes and autolysosomes were induced by tBHP in IFRS1 cells and a DHA pretreatment also reduced the production of tBHP-induced autophagosomes and autolysosomes.

Our observations were consistent with recent studies suggesting that hyperglycemia-induced oxidative stress induces cell death and autophagy [25,31,32]. Autophagy occurs at basal levels even under normal conditions and selectively processes aggregated proteins and damaged organelles. Autophagy plays a pivotal role in the physiological function of pancreatic β-cells. Pancreatic β-cells deficient in autophagy have shown an abnormal morphology and function of mitochondria and ER [33,34,35]. Autophagosome accumulations and the cell death of pancreatic β-cells in patients with type 2 diabetes indicate that autophagy may contribute to β-cell dysfunction and the development of diabetes [36]. Autophagy induced by hyperglycemia in podocytes [25] protects them from hyperglycemia-related apoptosis. On the contrary, another study reported that hyperglycemia decreased basal autophagy levels in podocytes [37]. In addition, it has been suggested that autophagy in neural tissue is a mechanism that eliminates stress-induced damage [38,39]. In cancer cells, on the other hand, autophagy can function to promote cell survival [40], but it can also induce cell death [41]. Therefore, autophagy exhibits both cell protection and cell damage, depending on the cellular environment. Taken together, the results of the oxidative stress-induced cell death and autophagy flux assay suggest that excessive oxidative stress activates autophagy and induces cell death in Schwann cells. For stresses below lethal levels, autophagy induction acts as a protective mechanism for cell survival. However, an excess of stress above a certain level induces apoptosis and cell death. For example, the over-activation of autophagy has been reported to induce apoptosis [42,43].

In particular, DHA elevates the intracellular ROS levels, induces autophagy, and promotes cell death in malignant cells [44]. Our previous study showed that DHA did not induce cell death or the formation of intracellular ROS in IMS32 cells [9]. Jing et al. [44] showed that DHA induced cancer cell death by inducing autophagy at a concentration of 50 μM. The DHA concentration in our experiments was 7.5 μM [9]. The contrasting findings in these studies may have resulted from different experimental conditions. High-dose DHA has been reported to increase the accumulation of p62 and cause autophagy [16,45]. However, in the present study, a pretreatment with 10 μM DHA for 12 h significantly decreased the LC3-II/LC3-I ratio, which was promoted by tBHP, and increased the p62 expression that was suppressed by tBHP (Figure 4a–c). These results demonstrated that autophagy induced by oxidative stress might be suppressed by a 7.5 μM DHA treatment. DHA induces the activity of antioxidant enzymes, such as catalase, glutathione peroxidase, and glutathione reductase [12]. DHA might protect Schwann cells by inducing antioxidant enzymes and suppressing autophagy enhancement due to excessive oxidative stress.

The p62 protein expression decreased in response to a treatment with 50 μM tBHP. As shown in Figure 3c, a DHA pretreatment increased the p62 protein expression, which was downregulated by a tBHP treatment; however, as shown in Figure 3c, chloroquine did not increase the p62 protein expression, which was again downregulated by tBHP. DHA effectively reduced TNF-α-induced necroptosis and autophagy in the L929 mouse fibrosarcoma cell line [17]. The receptor-interacting protein kinase 3 (RIPK3), a key molecule involved in necroptosis, inhibits the localization of LC3 and p62 complexes, indicating that RIPK3 functions as a negative regulator of selective autophagy [46]. Therefore, we speculated that these differences in necroptosis and autophagy pathways might have resulted in the difference in the p62 expression. However, it could not be clarified in this study. Future studies are required to clarify our speculations.

The AMPK signaling pathways act as signals to control cell metabolism, energy homeostasis, and cell growth and are recognized as central metabolic pathways activated by autophagy [47]. AMPK has been shown to promote autophagy by directly activating ULK1 through the phosphorylation of Ser317 during glucose starvation [48]. Our results showed that a pretreatment with DHA suppressed AMPK and ULK1 phosphorylation induced by oxidative stress. Oxidative stress-induced autophagy in IFRS1 cells might be dependent on the AMPK/ULK1 signaling pathway. Therefore, DHA might suppress autophagy activity, at least in part, by regulating AMPK activation in IFRS1 cells.

Previous studies have reported that alpha-lipoic acid, a natural antioxidant, protects against 6-OHDA-induced autophagy in SH-SY5Y cells [49]. DHA also suppresses TNF-α-induced necrosis and apoptosis in L929 murine fibrosarcoma cells [50] and human monocytic U937 cells [51]. Furthermore, DHA has been reported to antagonize TNF-α-induced necroptosis and autophagy in the L929 murine fibrosarcoma cell line [17]. These results clarified that DHA inhibited TNF-α-induced necroptosis and autophagy through the suppression of ROS [17]. We have previously shown that DHA suppressed ROS production caused by oxidative stress [9]; therefore, DHA might have suppressed autophagy and the necroptosis pathway. It is necessary to study other signaling pathways, such as necroptosis, in the future.

**Figure 7:**
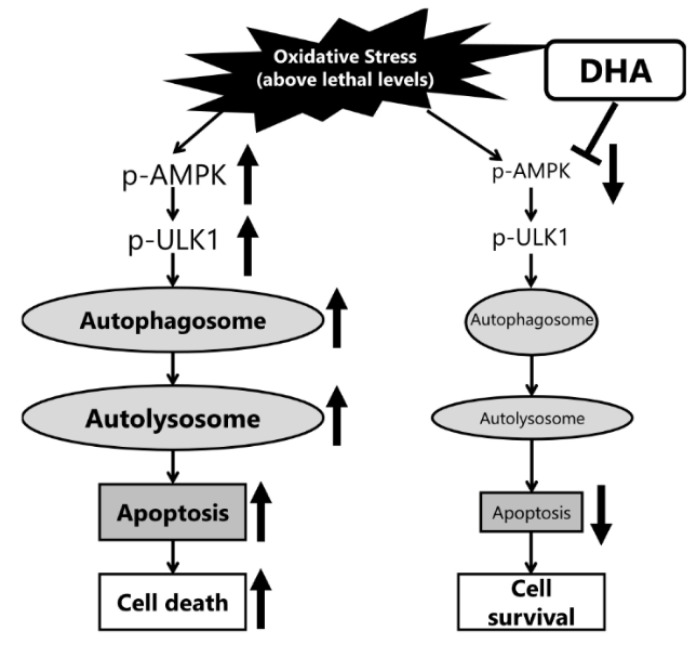
Schematic diagram illustrating the effect of docosahexaenoic acid (DHA) on autophagosomes in response to lethal levels of oxidative stress.

The autophagy pathway forms phagophores in the cytoplasm, then internalizes the organelles damaged by oxidative stress into autophagosomes. The degradation of the damaged organelles is performed with autolysosomes, generated by the fusion of autophagosomes and lysosomes [52]. Our findings indicated that oxidative stress induced the formation of autophagosomes and autolysosomes in IFRS1 cells (Figure 6). Furthermore, it has been shown that DHA is taken up through the cell membrane and changes the fluidity of the cell membrane. tBHP-induced autophagosome production and AMPK phosphorylation were completely suppressed by a DHA pretreatment; DHA has previously been reported to decrease the rate of autophagosome formation [53], suggesting that DHA might regulate autophagosome production via the AMPK signaling pathway. In the present study, DHA did not completely inhibit autophagy; autolysosomes were still present after a treatment with 50 μM tBHP, which may be potentially useful to further dismantle the deposition of aberrant material (Figure 7). The difference in the effects of DHA on the formation of autophagosomes and autolysosomes should be elucidated in future studies.

In the present study, we showed that DHA suppresses oxidative stress-induced autophagy and cell death via the AMPK-dependent signaling pathway in IFRS1 cells. Moreover, our findings suggested that a pretreatment with DHA might protect against diabetic neuropathy by ameliorating oxidative stress-induced autophagy and cell death in Schwann cells. Additional in vivo and clinical studies are needed to affirm the effects of DHA on diabetic neuropathy.

## 4. Materials and Methods

### 4.1. Materials

Fatty acid-free BSA was purchased from BBI Solutions (Cardiff, UK). DHA was purchased from Cayman (Ann Arbor, MI, USA). Dulbecco’s Modified Eagle Medium Nutrient Mixture F12 and fetal bovine serum (FBS) were obtained from Thermo Fisher Scientific (Waltham, MA, USA). Forskolin, tBHP, and MTT were purchased from Millipore-Sigma (St. Louis, MO, USA).

### 4.2. Cell Culture

An IFRS1 cell line was kindly gifted by Dr. Kazuhiko Watabe (Neurovirology Project, Department of Diseases and Infection, Tokyo Metropolitan Institute of Medical Science). IFRS1 cells were incubated with Dulbecco’s Modified Eagle Medium (DMEM) Nutrient Mixture F12 containing 5% FBS, antibiotics (100 units/mL penicillin and 100 μg/mL streptomycin), 20 ng/mL recombinant human heregulin-β (EMD Millipore, Temecula, CA, USA), and 5 μM forskolin (Millipore-Sigma, St. Louis, MO, USA). The cells were cultured in a humidified atmosphere containing 5% CO_2_ at 37 °C. The cells were subcultured every 2 d with 0.05% trypsin–EDTA (Millipore-Sigma, St. Louis, MO, USA) and reseeded into 6- and 96-well cell culture plates (Thermo Fisher Scientific, Waltham, MA, USA) and 8-well chamber slides (Watson, Kobe, Japan) at different cell densities. After culturing for 12 h in the medium described above, the cells were sustained in DMEM/F12 containing 1% FBS and an N2 supplement (Thermo Fisher Scientific, Waltham, MA, USA). The DHA (Cayman, Ann Arbor, MI, USA) was lysed in ethanol and gradually solubilized in a 2.6 mM fatty acid-free BSA solution. The BSA-conjugated fatty acids were dissolved in DMEM/F12 containing 1% FBS and an N2 supplement at the desired concentration. The control medium containing fatty acid-free BSA was prepared in a similar manner (the cells without DHA exposure, designated as the control, were incubated in a fatty acid-free BSA medium).

### 4.3. Cell Survival Assay

To identify the prophylactic effect of DHA on tBHP-induced cell death, IFRS1 cells were reseeded into 96-well plates (2 × 10^4^ cells/well) and cultured in a humidified atmosphere containing 5% CO_2_ at 37 °C for 12 h prior to treatment. The IFRS1 cells were pretreated with 10 μM DHA or 5 μM Compound C (Millipore-Sigma, St. Louis, MO, USA) for 12 h and exposed to 10–100 μM tBHP for 3 h. The cell viability was determined by MTT assays. After a treatment with tBHP, the cells were cultured with an MTT solution (0.5 mg/mL) in a culture medium for 3 h. The culture medium was discarded by suction and 200 μL DMSO was used to solubilize the formazan crystals. The absorbance at 570 nm was measured using a microplate reader (TECAN, Männedorf, Switzerland). The values were expressed as the percentage of cell viability. The absorbance of tBHP-untreated cells was set at 100%. The IFRS1 cells were reseeded into 6-well plates (2 × 10^5^ cells/well) and cultured in a humidified atmosphere containing 5% CO_2_ at 37 °C for 12 h prior to incubation. The IFRS1 cells were pretreated with 10 μM DHA for 12 h and exposed to 50 μM tBHP for 3 h. After incubation, the cell number was determined using a microscope (Olympus, Tokyo, Japan).

### 4.4. Measurement of Intracellular Reactive Oxygen Species (ROS)

The IFRS1 cells were reseeded into 8-well Ibidi μ-Slides (Ibidi, Grafelfing, Germany) (5 × 10^4^ cells/well) and cultured in a humidified atmosphere containing 5% CO_2_ at 37 °C for 12 h before a treatment to identify the prophylactic effect of DHA on tBHP-induced ROS production. The IFRS1 cells were pretreated with 10 μM DHA for 12 h. Intracellular ROS were measured using a ROS assay kit (Dojindo, Kumamoto, Japan). The DHA pretreated cells were washed with a Hanks Balanced Salt Solution (HBSS; FUJIFILM Wako Pure Chemical, Tokyo, Japan). A DCFH-DA working solution was added to the cells and incubated for 30 min, followed by a treatment with 50 μM tBHP or HBSS alone for 30 min. After washing with HBSS, the fluorescence intensity was measured by a fluorescence microscope (IX70; Olympus, Tokyo, Japan) and quantified using ImageJ software version 1.53m (NIH, Bethesda, MD, USA).

### 4.5. Western Blot Analysis

To identify the prophylactic effect of DHA on tBHP-induced autophagy, the IFRS1 cells were reseeded into 6-well plates (2 × 10^5^ cells/well) and cultured in a humidified atmosphere containing 5% CO_2_ at 37 °C for 12 h prior to incubation. The IFRS1 cells were pretreated with 10 μM DHA for 12 h and exposed to 50 μM tBHP for 3 h. The IFRS1 cells were lysed using a RIPA buffer with protease inhibitors (Millipore-Sigma, St. Louis, MO, USA). The protein samples were quantitated using bicinchoninic acid (BCA) reagents (Bio-Rad, Hercules, CA, USA) and heated in a loading buffer. The proteins (10 μg) were separated on 4–20% Mini-PROTEAN TGX gels and transferred onto a polyvinylidene fluoride membrane (Bio-Rad, Hercules, CA, USA). The membranes were blocked with 5% non-fat milk (Cell Signaling Technology, Danvers, MA, USA) in TBS-T (10 mM Tris-HCl, (pH 7.6), 100 mM NaCl, and 0.1% Tween 20) for 1 h at 25 °C and reacted with the primary antibody: LC3 (1:1000 dilution; MBL, Nagoya, Japan), p62 (1:1000; MBL, Nagoya, Japan), p-AMPK (Thr 172) (1:1000; Cell Signaling Technology, Danvers, MA, USA), AMPK (1:1000; Cell Signaling Technology, Danvers, MA, USA), p-ULK1 (Ser317) (1:1000; Cell Signaling Technology, Danvers, MA, USA), ULK1 (1:1000; Cell Signaling Technology, Danvers, MA, USA), Beclin1 (1:1000; MBL, Nagoya, Japan), or β-actin (1:5000; Millipore-Sigma, St. Louis, MO, USA) in Can Get Signal solution 1 (TOYOBO, Osaka, Japan) overnight at 4 °C. The membranes were washed with TBS-T three times and reacted with horseradish peroxidase-conjugated secondary antibodies (goat anti-mouse and goat anti-rabbit (Cell Signaling Technology, Danvers, MA, USA) for 1 h at 25 °C. The immunoreactive bands were visualized using a Clarity MAX Western ECL substrate (Bio-Rad, Hercules, CA, USA) and ImageQuant LAS-4000 (Cytiva, Tokyo, Japan). The relative densities of the bands were quantified using Image QuantTL software (FUJIFILM Corporation, Tokyo, Japan). The band densities were normalized using β-actin.

### 4.6. Autophagy Detection with DAPRed and DALGreen Staining

To identify the prophylactic effect of DHA on tBHP-induced autophagosomes and autolysosomes, the IFRS1 cells were plated onto 8-well chamber slides (Watson) (5 × 10^4^ cells/well) and cultured in a humidified atmosphere containing 5% CO_2_ at 37 °C for 12 h prior to a treatment. The IFRS1 cells were pretreated with 10 μM DHA for 12 h and cultured with 300 μL of 25 nM DAPRed (Dojindo, Kumamoto, Japan), 200 nM DALGreen (Dojindo, Kumamoto, Japan), and 1 μg/mL Hoechst 33342 (Thermo Fisher Scientific, Waltham, MA, USA) working solutions for 30 min. After the cells reacted with the reagents, they were washed twice with DMEM/F12 containing 1% FBS and an N2 supplement medium. The cells were cultured with 50 μM tBHP for 3 h then washed twice with a Live Cell Imaging Solution (Thermo Fisher Scientific, Waltham, MA, USA) and observed with a fluorescence microscope (BZ-X810) (Keyence, Osaka, Japan). The fluorescence images were analyzed using BZ-X800 Analyzer software version 1.1.1.8 (Keyence, Osaka, Japan).

### 4.7. Statistical Analysis

Data were expressed as a mean ± standard error for the indicated number of experiments. The statistical significance between the multiple groups was determined by a one-way analysis of variance (ANOVA) test and the Tukey–Kramer correction for multiple comparisons. *p*-values < 0.05 were considered to be statistically significant.
